# Hereditary Spastic Paraplegia: An Update

**DOI:** 10.3390/ijms23031697

**Published:** 2022-02-01

**Authors:** Arun Meyyazhagan, Antonio Orlacchio

**Affiliations:** 1 Dipartimento di Medicina e Chirurgia, Università di Perugia, 06132 Perugia, Italy; arun47biotech@gmail.com; 2 Laboratorio di Neurogenetica, Centro Europeo di Ricerca sul Cervello (CERC), Istituto di Ricovero e Cura a Carattere Scientifico (IRCCS) Fondazione Santa Lucia, 00143 Rome, Italy

**Keywords:** hereditary spastic paraplegia, neurodegenerative disease, neurogenetics

## Abstract

Hereditary spastic paraplegia (HSP) is a rare neurodegenerative disorder with the predominant clinical manifestation of spasticity in the lower extremities. HSP is categorised based on inheritance, the phenotypic characters, and the mode of molecular pathophysiology, with frequent degeneration in the axon of cervical and thoracic spinal cord’s lateral region, comprising the corticospinal routes. The prevalence ranges from 0.1 to 9.6 subjects per 100,000 reported around the globe. Though modern medical interventions help recognize and manage the disorder, the symptomatic measures remain below satisfaction. The present review assimilates the available data on HSP and lists down the chromosomes involved in its pathophysiology and the mutations observed in the respective genes on the chromosomes. It also sheds light on the treatment available along with the oral/intrathecal medications, physical therapies, and surgical interventions. Finally, we have discussed the related diagnostic techniques as well as the linked pharmacogenomics studies under future perspectives.

## 1. Introduction

Hereditary spastic paraplegias (HSPs) are a myriad of monogenic neurological defects aiding corticospinal and dorsal spinal cord axonal atrophy with a prevalence of 0.1–9.6 instances in every 100,000 around the world [1]. The critical manifestations include lower extremity bilateral spasticity, overactive reflexes, extensor plantar reflex, muscle fragility, and triggered gait deviations [2]. HSPs legacies are seen in almost all inheritance patterns, namely autosomal dominant (AD), autosomal recessive (AR), X-linked recessive (XLR), and mitochondrial, with about >80 susceptible gene loci registered to date [3,4]. They are clinically categorized as pure and complex, with the pure form characterized by neurologic impairment limited to progressive lower-extremity spastic weakness, hypertonic urinary bladder disturbance and mild diminution with vibration sensation.

A complicated form, also known as a complex form, is identified by the presence of other neurological or non-neurological manifestations such as seizures, dementia, muscle atrophy, ataxia, intellectual disability, peripheral neuropathy, extrapyramidal disturbance, gastroesophageal reflux, Dupuytren’s disease, or varicose veins (Table 1) [5,6,7,8,9,10,11]. HSPs can be triggered at infancy, toddling, puberty, or adulthood, with about 40% sporadic form [12].

Genetically, HSP categorization relies on the positions of causative genes with the designation “spastic paraplegia genes (SPGs)”, and personalized therapy is an unpopular solution, as treatment should target all the 80 genes. Thus, the current need is to find a therapy applicable for various genes involved in HSPs. Identifying the biomarkers, such as chromosomal locus at its earlier stage, can provide more insight into the disease pathophysiology and cellular propagation networks. The current review focuses on the chromosomes and the genes’ specific loci registered to date for HSPs and suggests possibilities for drug discoveries.

### HSPs Stratification

HSPs classification relies on (a) inheritance pattern (dominant, recessive, X-linked, mitochondrial, or maternal), (b) mutated gene, and (c) the clinical diagnostic syndromes involving symptoms/neurological findings (Table 2).

## 2. Autosomal Dominant (AD) HSP

**Figure 1:**
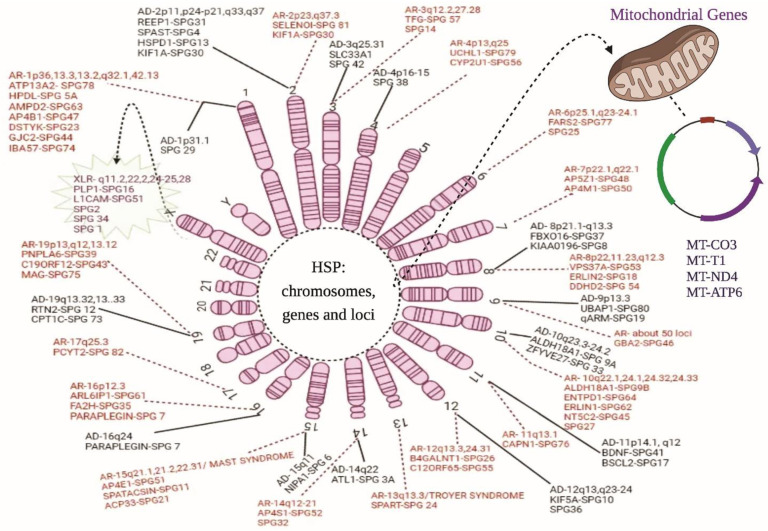
Chromosomal and genetic markers in HSP. The above diagram illustrates all the genes and their location on the chromosomes related to the HSP phenotypes: AD, autosomal dominant forms; AR, autosomal recessive forms; XLR, X-linked recessive forms; and mitochondrial inheritance. The solid line represents the AD forms of HSP, whereas the dotted lines show the AR forms. The spiky green cloud with a dotted line represents the XLR HSP form.

Autosomal dominant HSP is a highly prevalent inheritance form affecting 75–80% of registered cases with SPG4 (*SPAST* mutation) as the dominant phenotype [13]. The mean age of patients upon manifestation is 31.7, with an exception of 70 in a few cases with spasticity of the lower limbs along with/without bladder and sensory dysfunction [12]. The following chromosomal markers and genes (Table 3) are involved in AD HSP (Figure 1).

SPG3A is the second-most prevalent AD form, constituting 10% of registers cases, with the majority of them accounting for pure HSP and with prior onset by the age of 10 [14]; likewise, alterations in SPG6/*NIPA1* [15] and SPG8/*KIAA0196* [16] are linked with severe spasticity in the AD form. Mutation in the heat shock protein HSP60 (SPG13), SPG17/*BSCL2* are known to induce hereditary motor neuropathies [17]. Other prevalent AD forms of HSP have been observed due to mutations in the SPG31/*REEP1* [18] and SPG33/*ZFYVE27* genes [19].

### 2.1. Chromosome 1

Orlacchio et al. [8] found 1p31.1-p21.1 to actively associate with autosomal HSP transmission in a 19-member Scottish family with clinical presentations of sensorineural deafness, *pes cavus*, hiatus hernia, and hyperbilirubinemia by around the mean age of 15.2 and similarities such as haplotypes D1S2889 and D1S248.

### 2.2. Chromosome 2

A region such as that including p11, p24-p21, q33, and q37 is collated with HSP linkage by altering the receptor expression-enhancing protein-1 (SPG31/*REEP1*), spastin (SPG4/*SPAST*), Heat Shock Protein Family D (Hsp60) member 1 (SPG13/*HSPD1*), Kinesin Family Member 1A (SPG30/*KIF1A*) with frameshift [18], missense [20,21], and others including R11Q or S69L [22] in the heterozygous state to induce hyperreflexia plus spasticity and neuropathy.

### 2.3. Chromosome 3

Race-related heterozygous mutation in the Solute Carrier Family 33 Member 1 (SPG42/*SLC33A1*) in Chinese lineages with haploinsufficiency [23] at 3q25.31 shows classical signs of hyperreflexia, lower extremity muscular atrophy, and *pes cavus* at varying ages from 4 to 42 but predominately around 20 years. 

### 2.4. Chromosome 4

Orlacchio et al. [24] discovered a link between locus 4p16-p15 and spastic gait in people with mild to severe spasticity and distal end foot extensor weakness through genome-wide linkage analysis. 

### 2.5. Chromosome 8

Chromosome 8 is associated with characteristic forms of pathology, such as hyperactive reflexes of the upper limbs and muscle weakness of the iliopsoas with bladder disorders. These dysfunctions were observed in a large French family with an average age of 31.6 years by Hanein et al. [25]. The disease locus 8p21.1-q13.3 was discovered as a pathological region for SPG37. Analysing muscle atrophy biomarkers such as F-box proteins (F-box protein 16 (*FBXO16*) at p21.1) can shed more light on the respective gene involvement in HSP pathology [26]. Another mutation around SPG8/*KIAA0196* at the 8q24 region contributed to milder and purer HSP discovered in a North American family by Valdmanis et al. [16].

### 2.6. Chromosome 9

Ubiquitin-associated protein 1 (*UBAP-1*) for endosome transportation-1 (*ESCRT-I*) to regulate vesicle trafficking is located at 9p13.3 l and associated with a complex sorting mechanism. *UBAP1* degrades ubiquitin surface proteins [27]. Nonsense or frameshift heterozygous mutations in SPG80/*UBAP1* induce AD by truncating protein in the UMA domain of the N-terminal. Studies by Lin et al. [28] showed allele variants of *UBAP1* such as 526G-T transversion. In various ethnic groups, 8-bp insertion in the 4th exon showed the phenotype of early exosome deletion, clustering, enlargement and ubiquitinated protein accumulation in the cytoplasm of HeLa cells and wildtype cortical nerve cell culture in the mouse model. These alterations can reduce the length of motor neurons or change the shape of axons. Valente et al. [29] identified another autosomal dominant form of HSP on chromosome 9 in its q arm among an Italian family of 10 members with the disease LOD of 3.31 and symptoms of abnormality in gait, ankle and knee colnus, and urine urgency. The researchers designated it as SPG19.

### 2.7. Chromosome 10

Studies by Coutelier et al. [30] and Seri et al. [31] showed 10q23.3-q24.2 with heterozygous mutation in Aldehyde Dehydrogenase 18 Family member A1 (SPG9A/*ALDH18A1*) is linked to spinocerebellar ataxia with least expressed features such as cataract and dysarthria in people, causing the onset to occur anywhere from adolescence to adulthood. Another study by Mannan et al. [32] showed that chromosomal region 10q24.2 contains the FYVE27-type Zinc Finger gene in a susceptible region (SPG33/*ZFYVE27*) and the related protein interacts with spastin, essential for maintaining axonal growth of motor nerves in the spinal cord [33]. On the other hand Martignoni et al. [19] raised significant doubts as to whether the ZFYVE27 gene may be the cause of SPG33.

### 2.8. Chromosome 11

Zhao et al. [34] saw AD HSP SPG41 in a Chinese family with an average onset age of 16.6 years followed by weakness in lower limbs plus gait spasticity and hyperreflexia (2.36 LOD). Another heterozygous mutation in 11q12—Berardinelli–Seip congenital lipodystrophy 2 (SPG17/*BSCL2*)—is responsible for seipin production to maintain adipocytes and metabolism in HSPs phenotypes with weakness and clawed lower limbs, sensory impairment, and occasionally tremors as characteristics [35]. 

### 2.9. Chromosome 12

A heterozygous mutation at the 12q13 kinesin-5A (SPG10/*KIF5A*) is necessary for moving macromolecules and organelles inside the cells, i.e., axonal transport is linked with AD neurological disorders forms such as spastic paraplegia and peripheral neuropathy [36,37]. In addition, 12q23-q24 locus induces HSP phenotype with a disease LOD score of 3.23 and a mean onset age of 24 years. The individuals were shown to have spasticity, gait, and extremity weakness, with extensor plantar response [38]. 

### 2.10. Chromosome 14

Heterozygous mutation of atlastin-1 (SPG3A/*ATL1*) at q22 correlates with almost 10% of registered AD HSPs with pathological alterations such as axonal degeneration in the vertebral column corticospinal region [39]. The gene encodes the GTPase dynamic region involved in forming networks of endoplasmic reticulum (ER) tubules and elongates the axons of neurons [40].

### 2.11. Chromosome 15

Gene SPG6/*NIPA1* seen at 15q11 is required for the proper functioning of ER [41], and its mutation is associated with ankle dorsiflexion and hip flexion debility as marked lower limbs spasticity.

### 2.12. Chromosome 16

A heterozygous mutation in SPG7/*PGN* gene at 16q24 is characterised by extensive weakness and spasticity in lower extremities due to axonal degeneration [42]. The gene produces ATPase associated with various cellular activities (AAA) protein family protein paraplegin, required for cellular activities, along with axon regeneration [43].

### 2.13. Chromosome 19

Reticulon 2 (SPG12/*RTN2*) at 19q13.32 produces protein coding for shaping ER proteins; complete deletion/frameshift mutation of the gene results in the production of truncated proteins, causing improper interaction between spastin and ER localisation [44]. Van De Warrenburg et al. [45] showed that *RTN2* haploinsufficiency is enough to induce HSP characteristics. Rinaldi and his group in [46] reported 19q13.33 to induce HSP phenotype due to carnitine palmitoyl-transferase 1C (SPG73/*CPT1C*) and alterations that modify lipids signal transduction for disease pathology.

## 3. Autosomal Recessive (AR) HSPs

AR HSPs are rare and confined to single families or single persons with heterogeneity and an ever-growing list of recently observed genes. Consanguineous marriage increases the frequency of AR HSPs in a given community, and about <30% of the registered HSPs cases show recessive inheritance patterns. Usually, the phenotypic characters are different between each family member but are complex invariably [1]. SPG11 is the most prevalent form of AR HSPs seen in about 8% of the registered cases ranging from 4 to 36 years of age. The instances with AR pattern usually show cases with learning disabilities and spasticity in the lower extremes in the second decade of the patient’s life. Table 3 lists out the chromosomes, their locus, and genes involved in the AR pattern of HSP. About 50% show neuropathy of motor axons, ataxia, dysarthria, progressive spasticity in the upper body, and visual failure [47]. Below, the chromosomal markers linked to AR pattern are discussed with their loci and the gene involved in the HSPs progression (Figure 1).

### 3.1. Chromosome 1

A homozygous or compound heterozygous alteration of ATPase cation transporting 13A2 (SPG78/*ATP13A2*) at 1p36 locus is responsible for lysosomal enzymes necessary for transporting inorganic cations via endo-lysosome cargos and maintaining neurons integrity [48]. The alteration culminates in an AR onset in patients born to consanguineous marriage with clinical manifestations including gait abnormality, lower extremity weakness, hyperreflexia, and a few shows of bladder neurological dysfunction [49].

Husain et al. [50] identified another AR HSP due to compound heterozygous mutation (missense) in 4-hydroxyphenylpyruate dioxygenase (SPG5A/*HPDL*) at 1p34.1 region comprising the conserved pattern of 371 amino acids across vertebrates. The gene’s function is uncertain, though the clinical manifestations include delayed neural development, with lower limbs spasticity. Few other regions such as 1p13.3, 1p13.2, 1q32.1, 1q42.13, and 1q42.13 have been identified to induce HSPs due to heterozygous compound alterations in the Adenosine Monophosphate Deaminase 2 (SPG63/*AMPD2*) [51], Adaptor Related Protein Complex 4 Subunit Beta 1 (SPG47/*AP4B1*) [52], Dual Serine/Threonine and Tyrosine Protein Kinase (SPG23/*DSTYK*) [53], Gap Junction Protein Gamma 2 (SPG44/*GJC2*) [54], and Iron-Sulfur Cluster Assembly Factor (SPG74/*IBA57*) [55].

### 3.2. Chromosome 2

An AR HSP is seen in some patients with selenoprotein I (SPG81/*SELENOI*) mutations at 2p23 locus with clinical manifestation of delayed motor neuron development and spasticity, with neurological disabilities including speech delay and reduced intellect, microcephaly, seizures, and ocular abnormalities [56]. The protein translated from the *SELENOI* consists of rare selenocysteine encoded to the UGA codon to terminate the translation signals, normally known as Choline/ethanolamine phosphotransferase (*EPT1*), necessary for myelination and neural development. Similarly, another pathological locus at 2q37.3 is Kinesin Family Member 1A (SPG30/*KIF1A*). This region follows both AR and AD patterns in pure and complicated HSPs [57], as it encodes for anterograde motor protein needed for transporting organelles with membranes in the axon microtubules. 

### 3.3. Chromosome 3

Through genome sequencing, Slosarek et al. [58] identified a mutation in tropomyosin receptor kinase fuse (SPG57/*TFG*) at 3q12.2 that induces HSP characters in a recessive pattern. The gene maintains integrity in the premature secretory route [59], and its alteration leads to ER stress, culminating in destroying axons’ self-associating ability. Likewise, the homozygosity at 3q27 to 28 regions shows a multipoint LOD score of 3.9 and is designated as SPG14 [60], as it hosts genes associated with neuronal development, specifically Small Ubiquitin-like modifier (SUMO) specific peptidase 2 (SPG14/*SENP2*); it might contribute to the pathology and aids in dendritic spine modification necessary for neuronal activity in excitatory synaptic mechanism [61].

### 3.4. Chromosome 4

Two sites on chromosome 4 are linked with AR HSPs. Homozygous or compound heterozygous mutations in the neuronal gene Ubiquitin C-terminal Hydrolase L1 (SPG79/*UCHL1*) at 4p13 contribute to neurodegenerative phenotypes [62], with clinical manifestations including cognitive deterioration with neuropathy in the peripheral region and cerebellar ataxia [63]. Parallelly, a mutation in the Cytochrome P450 Family 2 Subfamily U member 1 (SPG56/*CYP2U1*) at 4q25 leads to early onset of spasticity in the lower body parts, aiding in walking disabilities by inhibiting the P450 hydroxylase enzyme activities, causing loss of protein structure, leading to ataxia, retina impairment, and neuropathy [64].

### 3.5. Chromosome 6

Homozygous or compound heterozygous mutation in the phenylalanyl-t-RNA synthetase 2 (SPG77/*FARS2*) at p25.1 is associated with a recessive form characteristic dysfunction in mitochondria, development postponement, and seizures since childhood [65]. The *FARS2* transfers amino acid to its cognate mitochondrial tRNA, and its variants are linked with encephalopathy with epilepsy and other spastic paraplegia symptoms [66]. Another hotspot, 6q23-q24.1, is linked with AR HSP. Zortea et al. [67] found a LOD score of 3.28 in this region in spastic paraplegia of Italian families with consanguinity. They suggested a detailed analysis of the connective tissue growth factor/cellular communication network 2 (SPG25/*CNN2*) at 6q23.2 has elevated *CNN2* protein, thereby inducing neuromuscular pathologies such as neurodegenerative disorders, muscular dystrophies, and muscle overuse [68].

### 3.6. Chromosome 7

Two loci, 7p22.1 and 7q22.1, are associated with AR HSPs, as Hirst et al. [69] and Tüysüz et al. [70] described. The 7p22.1 mutation in Adaptor Related Protein Complex 5 Subunit Zeta 1 (SPG48/*AP5Z1*), facilitating intracellular transmembrane proteins sorting and trafficking cargos through vesicles, results in motor and sensory neurons neuropathy, ataxia, parkinsonism, and cognitive impairments [71]. In contrast, a mutation in the Adaptor Protein complex-4 Mu 1 subunit (SPG50/*AP4M1*) regulating vesicular trafficking in endocytic ways recruiting and selecting cargo proteins at 7q22.1 results in loss of function and declines the production of mature neurons and may cause microcephalies with short statures [72].

### 3.7. Chromosome 8

Four loci associated with AR HSPs are designated as SPG53 (8p22), SPG18 (8p11.23), SPG54 (8p11.23), and SPG5A (8q12.3). Homozygous or compound mutation in the Vacuolar protein sorting-associated protein 37A (SPG53/*VPS37A*) required in grouping ubiquitinated transmembrane proteins of multivesicular bodies into internal vesicles (Zivony-Elboum et al. [73] can delay motor neuron development in the initial two years of life to cause lower limbs spasticity, speech delay, and cognitive disabilities. Similarly, alteration in the Endoplasmic Reticulum Lipid Raft-Associated 2 (SPG18/*ERLIN2*) is necessary for degrading ER protein via ubiquitin-proteasome and calcium signalling in lipid synthesis, resulting in neurological symptoms with lower limbs weakness and spasticity [74]. Alteration in the phospholipase gene SPG54/*DDHD2* at p11.23 induces the HSP phenotype at early ages, with intellectual disability and white matter abnormalities observed in scans [75]. One more mutation at q12.3 leads to neurodegenerative phenotype due to loss of function of the cytochrome P450 oxysterol 7 α-hydroxylase (*CYP7B1*) coding gene, producing hydroxycholesterols substrates in plasma and cerebral fluids of HSP patients instead of bile. Increased substrates impair the viability and metabolic routes of pluripotent stem cells of cortical neuronal regions [76].

### 3.8. Chromosome 9

A recessive HSP characterised by lower limbs weakness, spasticity, declined brain development, bilateral congenital cataract, jaw jerks, and tendons reflexes is observed due to mutations in the non-lysosomal glucosylceramidase β2 (SPG46/*GBA2*) located in the ER and Golgi apparatus membrane to cleave glucosylceramide located in neurons of cortex region at higher concentrations. Usually, nonsense or missense mutation is observed with loss of function of *GBA2* in SPG46 patients [77].

### 3.9. Chromosome 10

In 2004 Meijer reported 10q22.1-q24.1 region is associated with the HSP phenotype due to homozygous mutation, and through atlasgeneticsoncology.org (accessed on 15 October 2021), we discovered the adenosine kinase (SPG27/*ADK*) is located in the region, and focused study on this gene might elucidate HSP pathophysiology. As *ADK* primarily assess the adenosine levels at synapses, an increased level of adenosine is implicated in epilepsy pathogenesis [78]. Similarly, Aldehyde Dehydrogenase 18 Family Member A1 (SPG9B/*ALDH18A1*) and Ectonucleoside triphosphate diphosphohydrolase 1 (SPG64/*ENTPD1*) at q24.1 are shown to cause AR HSP [30,51]. Other loci q24.31 and q24.32-33 are also linked with HSP phenotypes due to mutations in ER lipid raft Associated 1 (SPG62/*ERLIN1*) and 5′-Nucleotidase, cytosolic II (SPG45/*NT5C2*), either in a homozygous or compound heterozygous state [51].

### 3.10. Chromosome 11

Calpain 1 (*CAPN1*) at 11q13.1 is studied widely due to its universal presence in various tissues and organs, and its predominant role is protecting and maintaining neurons plasticity in the synaptic regions and alterations comprehend to various brain disorders [79]. Through animal models, Gan-Or et al. [80] observed that loss of function of the *CAPN1* leads to locomotive organ defect, axonal abnormalities, and negative geotaxis based on age.

### 3.11. Chromosome 12

SPG26/*B4GALNT1* [81] and mitochondrial DNA for oxidative phosphorylation mitoribosomes (SPG55/*C12ORF65*) [82] are found at 12q13.3 and q24.31. Mutations in these genes are linked with peripheral neuropathies, optic zone atrophy, cognitive disabilities, and pyramidal characteristics.

### 3.12. Chromosome 13

Homozygous or compound heterozygous mutation of Strumpellin and Spartin proteins necessary for axonal integrity maintenance at locus 13.3q induces Troyer syndrome, an HSP form SPG20/*SPART* [83] with ankle clonus, atrophy of the cerebellar vermis, loss of white matter volume, skeletal abnormalities with high arched feet, muscular weakness, and hyperreflexia with oromotor dysfunction [84]. Likewise, Hodgkinson et al. [85] showed paraplegia linkage SPG24 around the 13q14 in contributing spasticity, clonus, and a scissoring gait.

### 3.13. Chromosome 14

Linkage analysis studies revealed the 14q12 region as the prime region to induce SPG32 HSP phenotypes in consanguineous marriage families around 24 years of age with clinical manifestations including autonomic dysfunction features in the legs, with weakness, extensor plantar responses, and mental retardation [86]. Two scientists independently observed homozygous and compound heterozygous mutation in adaptor-related protein complex 4 subunit sigma 1 (SPG52/*AP4S1*) at 14q21 in Syrian [87] and Caucasian families [88], which induces HSP phenotypes due to defective endosomes trafficking. Maemoto et al. [89] showed that *DDHD1* protein depletion negatively affects neurite growth due to defective endosomal protein recruitment other than tubular recycling endosome. Another gene, Zinc Finger FYVE-Type Containing 26 (SPG15/*ZFYVE26*) at 14q24.1, is linked with [90] spatizin, and its mutations lead to HSPs. Furthermore, 14q32.31 contains a potential HSP pathogenic variant, the tectonic beta-propeller repeat-containing 2 (SPG49/*TECPR2*) necessary for degrading proteins (autophagy) to maintain the cellular viability and homeostasis, and discrepancy in this mechanism is the foundation for a wide range of muscular and neurodegenerative disorders, including HSP [91].

### 3.14. Chromosome 15

Biallelic mutations at 15q21.1, 15q21.2, and 15q22.31 are associated with paraplegia-like characteristics in HSP individuals. The genes involved include SPG11/spatacsin encoding gene, Adaptor Related Protein Complex 4 Subunit Epsilon 1 (SPG51/*AP4E1*), and Acidic Cluster Protein 33 (SPG21/*ACP33*). SPG11 is seen along with parkinsonism and upper body neuron spastic phenotypes [92]. *AP4E1* plays an important role in trafficking proteins intracellularly, and its alteration culminates in microcephaly, cognitive impairment, and seizures, with depleting corpus callosum and limb stiffness [93]. Alteration in *ACP33* encoding maspardin leads to a recessive form of paraplegia and intellectual impairment with cerebellar atrophy [94].

### 3.15. Chromosome 16

The ADP-ribosylation factor-like 6 interacting protein 1 (SPG61/*ARL6IP1*) at 16p12.3 stabilizes and produces intensely curved tubular membranes to maintain ER integrity and structure [95]. In addition, two more HSP susceptive regions are found at 16q23.1 (SPG35/*FA2H*) and 16q24.3 (SPG7/*PGN*). The *FA2H* produces the FA2H protein incorporated in the ceramide group for myelin production, and mutations lead to neurodegenerative disorders, with higher iron residues in the brain [96]. Likewise, alteration in the paraplegin leads to mitochondrial disorganization and increased aggregation, making it a hotspot to induce clinical manifestations such as ataxia, optic neuropathy, tremors, and gait spasticity [97].

### 3.16. Chromosome 17

Vaz et al. [98] showed the gene at 17q25.3 is associated with muscular spasticity. The phosphate cytidylyltransferase 2, ethanolamine (SPG82/*PCYT2*) is involved in the complex lipid metabolism, and its mutations lead to HSP phenotypes, as *PCYT2* encodes phosphoethanolamine cytidylyltransferase, playing a significant role in the CDP-ethanolamine pathway (Kennedy pathway) for phosphatidylethanolamine (PE) production. PE with phosphatidylcholine (PC) comprises human membrane phospholipids [99].

### 3.17. Chromosome 19

Chromosome 19 embody three regions p13, q12, and q13.12 linked with AR HSP with homozygous/complex heterozygous alteration in Patatin-Like Phospholipase Domain Containing 6 (SPG39/*PNPLA6*), Chromosome 19 Open Reading Frame 12 (SPG43/*C19ORF12*), and Myelin Associated Glycoprotein (SPG75/*MAG*), respectively. The PNPLA6 hydrolases phosphatidylcholine (PC) and lysophosphatidylcholine (LPC) required for ER synthesis are linked to various malaise conditions due to disruptive effects. Hence, a mutation in *PNPLA6* jeopardizes lipid homeostasis responsible for diseases disturbing the central and peripheral nervous system plus neuron health [100]. Mutation of *C19ORF12* induces spasticity and weakness confined to lower extremities, with skin peeling and declining intellect [101]. MAG proteins expressed by myelination cells and are designated as Siglec-4a (sialic acid-binding immunoglobulin type lectin) necessary for axon attachment with glial cells, axon regenerating, neurites outgrowth inhibition, and neuron protection from axonal damage and any missense variations lead to oculomotor apraxia, neuropathy ataxia [102].

## 4. X-Linked Recessive (XLR) HSPs

### Chromosome X

About 1–2% of HSP candidates follow X-linked inheritance patterns in sporadic genetic forms. This HSP shows a distinct phenotype of pure and complicated with spasticity and weakness in a slow progressive way in the lower legs in pure form, whereas dementia, ataxia, retinopathy, epilepsy, ichthyosis are observed in complicated cases. About four loci have been registered to date to induce HSP characteristics on the X chromosome (Figure 1). The loci q11.2, q22.2, q24-25, and q28 are SPG16, SPG2, SPG34, and SPG1, respectively (Table 3). 

Mutation in proteolipid protein (*PLP1*) at q22.2 induces lower extremity spasticity severely. Animal and cell transfection models showed oligodendrocyte apoptosis and PLP accumulation in ER, leading to unfolded protein response causing HSP characteristics [103]. 

The usual brain function is controlled by the temporal and structural association between the cells with L1 cell adhesion molecules members (SPG1/*L1CAM*) through hetero- and homophilic interactions. Any alterations of q28 in *L1CAM* (usually loss of function) lead to development abnormalities, with cognitive impairment, limbs spasticity, corpus callosum aplasia, axons outgrowth, hydrocephalus, and myelination anomalies collectively known as CRASH or L1 syndrome [104].

## 5. Maternal Inheritance (Mitochondrial HSPs)

Being sporadic, maternal inheritance is mainly seen in complex HSPs, primarily directing the focus towards mitochondrial disabilities. These are the rarest HSP kinds and affect nearly 1–2% of HSP cases. Usually, alterations linked to *ATP6* gene’s “m.9176 T>C” are associated with mitochondrial function impairment for the HSP phenotype. Similar alteration is observed in Leigh syndrome, implicating the role of modifying environmental and lifestyle factors to express various phenotypes for the same alteration [105]. Mutations in *MT-CO3* gene encoding for Cytochrome c oxidase III/complex IV—respiratory chain complex IV subunit; *MT-T1* gene associated with Isoleucine transfer RNA; and *MT-ND4* and *MT-ATP6* genes encoding for Complex V, ATP synthase, and subunit ATPase 6—respiratory chain complex V subunit are shown to impart mental retardation, cerebellar ataxias, loss of hearing, chronic progressive external ophthalmoplegia, and neuropathy in few rare HSPs [106].

### 5.1. Predictive Genes for the Various HSP Forms:

#### 5.1.1. Chromosome 1 

Through an atlasgeneticsoncology.org (accessed on 15 October 2021) neuronal growth regulator-1 (SPG29/*NEGR1*) is seen in the 1p31.1 region and other multiple genes, and region 1p31.1-21.1 is linked with AD SPG29, according to Orlacchio et al. [8]. Future study can involve special investigation around the *NERG1,* which might help explain the pathology of HSPs.

#### 5.1.2. Chromosome 4

Based on studies by Orlacchio et al. [9], locus 4p16-p15 is linked with AD SPG38 as the Janus kinase, and microtubule interacting protein 1 (*JAKM1P1*) is at p16.1, and it plays an important role in spinal cord development, neuronal polarity balance as axons growth and apoptosis [107]. Thus, the study of this gene might shed light on the pathology of HSP.

#### 5.1.3. Chromosome 11

Mellesmoen et al. [108] showed that applying brain-derived neurotrophic factor (*BDNF*) postpones cerebellar dysfunction endogenously in ataxia mouse models, which can explain a similar mechanism for cognitive slippage in complicated HSPs forms (SPG41), as *BDNF* is at 11p14.1.

#### 5.1.4. Chromosome 12

The locus of 12q23-q24 induces HSP phenotype (SPG36) with a disease LOD score of 3.23 along with the phenotypes of spasticity, gait, extremity weakness, and extensor plantar response [38]. Since cytoskeleton-associated protein 4 (*CKAP4*) is located at q23.3, it can contribute to the pathology, as it is a cytoskeletal protein required for the microtubules formation and signals transduction along with axonal guidance and synapses formation [109].

#### 5.1.5. Chromosome X

The detailed insight in the myotubularin-related protein 8 (*MTMR8*) and zinc finger-type containing-C4H2 (*ZC4H2*) at q11.2 regions might provide details about the gene involved in the pathogenesis of SPG16, as studies on zebrafish showed the role of *MTMR8* in regulating actin filament modelling necessary for muscular and vascular development [110]. Similarly, deficiency or mutation associated with *ZC4H2* is linked with hyperreflexia, arthrogryposis, and muscle weakness [111]. Glutamate metabolism is primitive for the brain’s normal functioning and signal transmission. Glutamate dehydrogenase is expressed in astrocytes and affects the glutamate and tricarboxylic acid metabolic cycle to cause neurodegenerative disorders such as Parkinson’s. Hence, we suggest that shedding more effort in the glutamate dehydrogenase 2 (GLUD2) at q24 on the X chromosome can strengthen SPG34 pathophysiology understanding [112].

## 6. Neuron’s Pathology in HSP 

Lateral corticospinal axonal degradation is predominantly observed in HSP postmortem studies with a higher depletion rate in the spinal cord’s thoracic zone distal end and cervical zone mainly due to axons degeneration in fasciculus gracilis fibres to demyelinating [37]. Sometimes, degeneration can extend to the rostrum, internal capsule, peduncles of the cerebellum, pons, and medullary zone, with declined Betz cells concentration (pyramidal neurons) [17].

**Figure 2:**
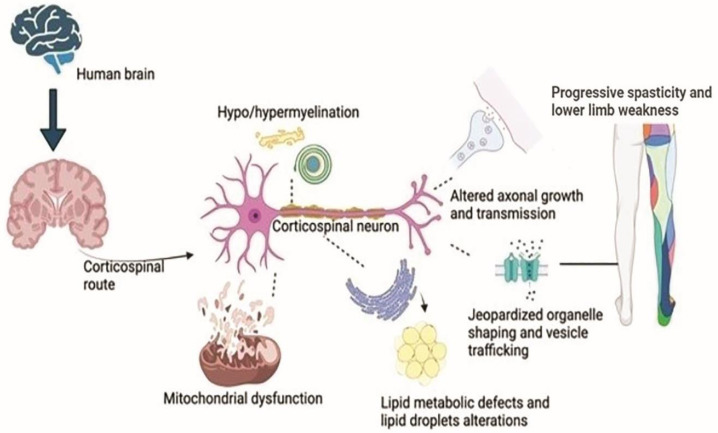
Biological dysfunction in HSP. The neurons in the corticospinal zone of the brain undergo mutations in the genes, causing a breakdown of organelle shaping and trafficking and dysfunction in the mitochondrial cells at the neuron’s nuclear region. Few gene mutations lead to faulty transmission in the axons, and some mutations cause degeneration of the myelin sheath of the corticospinal neuron. Likewise, an endoplasmic reticulum shaping genes’ mutation causes defective metabolism, especially lipid droplet formations. All these characteristics lead to lower limb spasticity and weakness, causing HSP phenotypes.

The central nervous system (CNS) long axons are hotspots and the first site of HSP axonopathy. Peripheral neuropathy, a common HSP subgroup symptom, is caused by the depletion of other neurons. Due to neuropathy in specific locations, shorter neurons in the basal ganglia, cerebellum, anterior horn cells, and Clarke’s column cause HSP features. [113]. Neuronal region genetic mutations cause deformities predominantly in the myelin layer, and other studies revealed cerebellar atrophy and CNS myelination, along with corticospinal axonal degeneration and developmental disorders, smaller spinal cord diameter, and thin corpus callosum as classical developmental abnormality signs in HSPs [106] (Figure 2).

## 7. Epidemiology of the HSP Genes

With the global prevalence of 0.1–9.6 in every 100,000 individuals, based on geographical location [114], HSP is mainly observed in AD pure form in about 80% of the North American and north European HSP populations, with SPG4/*SPAST* mutations in 40%, SPG3A/*ATL1* mutations in 10% at HSPs’ early beginning, about 10% SPG31/*REEP1* mutations, and almost 3% SPG10/*KIF5A* mutations [114,115]. Markedly, the mutation mentioned above is seen in complex HSPs and other neuropathies involving motor and sensory neurons [116].

On the other hand, AR HSPs are more complex and seen in a high degree of the consanguineous marriages population, with nearly 30% registered HSPs from the Middle East and northern Africa. SPG11 and SPG15 constitute a significant chunk of AR forms of HSPs [117]. The common phenotypic characters include thinning corpus callosum, periventricular white matter change in the ear lynx, early development of parkinsonism, cognitive ability slacking, moderate ataxia, retina abnormalities, and prominent paraplegia [114]. In addition, SPG35/*FA2H* and SPG45/*C19orf12* [51,118] are prevalent forms of AR. SPG5A shows *CYP7B1* pathological variant in 7.3% recessive form and 3% sporadic pure form [119]. Likewise, 5–12% of cases account for AR SPG7 [120], and about 3–5% of HSP individuals show SPG11 variants in AR mode.

XLR HSPs shows complex phenotypes with few cases, and five HSPs are known to date with three genes identified as SPG22/*SLC16A2*, SPG1/*L1CAM*, and SPG2/*PLP1*.

Late spastic paraplegia-like symptoms are due to alterations (m.9176T>C) in the *ATP6* of mitochondrial DNA [121]. Similarly, with their colleagues, Sánchez-Ferrero et al. [122] reported alterations in mitochondrial *MT-CO3* and *MT-T1* causing HSP. About 1–2% of cases show X and mitochondrial chromosomes mutations. 

## 8. The Typical Cellular Models in HSP Pathogenesis

Genetic forms of HSP causes colossal confusion, and for better understanding, the end products of genes are grouped based on pathogenic concern at cell levels (Table 4).

## 9. Organelles and Membrane Morphogenesis

Characteristics such as intracellular distribution, shaping membranes, trafficking, polarity biogenesis, and long axons in corticospinal seen in HSPs involve mutations in the principal genes AD HSP (SPG4, SPG3A, and SPG31) and AR HSP (SPG11 and SPG15).

### 9.1. An ATPase Integrated with Microtubule and Membrane Structure—Spastin

Spastin isoforms are translated from the SPG4/*SPAST*, and any mutation is linked with two isoforms of spastin, and its deficiency impairs axonal branching and microtubules maintenance [114]. SPG5A shows *CYP7B1* pathological variant in 7.3% recessive form and 3% sporadic pure form [119]. Likewise, 5–12% of cases account for AR SPG7 [120].

### 9.2. A Big GTPase Amalgamating ER Tubules—Atlastin

Atlastin GTPase fuses similar ER tubules to give polygonal structure in the periphery, and Atlastin deletion impairs axons elongation in forebrain neurons [123] in SPG3A cases. Basically, atlastin-1 is observed in the growth of neurites and helps in intracellular membrane trafficking at the interface between Golgi and ER; additionally, Atlastin functions significantly in the morphogenesis of ER and Golgi [17].

### 9.3. Maintaining ER Morphology

Proteins such as spastinM1, atlastin-1, REEP1, RTN2, ARL6IP1, RAB3GAP2, Protrudin, and REEP2 [114,115] retain the structure and functioning of the ER tubules. 

### 9.4. Defects in Metabolism of Lipid and Its Droplet

ER impairment jeopardizes lipids and sterols metabolism, synthesis, and distribution as lipid droplets (LDs) for fat storage. SPART HSP pathophysiology is modulated by LD biogenesis due to alteration of AIP4 (atrophin-1 interacting protein 4) to ubiquitinate proteins of LD [124]. Indirectly, proteins such as SLC33A1 lead to the lipids and sterols biosynthetic pathway. 

### 9.5. Lysosomal Regeneration by Spatacsin and Spastizin

Spatacsin and spastizin proteins bind with each other and function simultaneously to generate lysosomes for autophagy to adapt to environmental changes, and their dysfunction leads to HSP pathology [125].

### 9.6. HSP Adaptor Proteins

Alterations in adaptor protein complexes (AP-4) for trafficking amyloid precursor protein from trans-Golgi apparatus to endosomes leads to SPG47, SPG50, and SPG52 [87].

### 9.7. Endosome Tubule Formation and Strumpellin

Endosome shape and peddling alteration seen in HSP phenotype are due to a mutation in strumpellin protein and the core of WASH complex’s necessary for organising cytoskeleton and membrane modelling HSPs [126].

### 9.8. Organelles Shape Defects Inducing Axon Pathology

Membrane modelling proteins such as Spastin M1, REEP1, Atlastin, spastizin, spatacsin, and strumpellin from ER, endosomes, and lysosomes induce pathological states, of which further research is required.

## 10. Signalling of Bone Morphogenetic Protein (BMP)

HSP proteins such as NIPA1, atlastin-1, and spastin inhibit BMP signalling leads to HSP phenotypes such as SPG3A and SPG4 [114]. 

## 11. Transport through Motor Proteins

Mutations in motor proteins such as KIF5A KIF1C and KIF1A leads to HSP by impairing vesicle transmission between the Golgi body and ER [115].

## 12. Mitochondrial Dysfunction

Alterations in mitochondrial genes including SPG7/*PGN* and SPG13/*HSP60* induce HSPs phenotypes, and AR HSP form has been seen due to loss of mitochondrial *IBA57* (Iron-Sulfur Cluster Assembly) and translation proteins of mitochondria as SPG74 and SPG77 [55]. Spartin is located in mitochondria, and its alteration causes SPG20 [114].

## 13. Path of Axon

Loss of function of L1CAM is linked with abnormal corticospinal tract development. L1CAM links to neuropilin-1 to counteract with semaphorin 3A protein plexin for guiding corticospinal neurons cells, and its mutations hinder axons traversing in the midline region, inducing SPG1 phenotype [127].

## 14. Defects in the Myelination Process (Hypo or Dysmyelination)

CNS dysmyelination is common in spastic paraplegia conditions such as inherited leukodystrophies and multiple sclerosis. PLP1 encoding DM20 alters 50% of the myelin sheath, and its deletion causes SPG2. Likewise, GJC2 producing connexin 47 (CX47) alteration causes SPG44, and MAG (myelin-associated glycoprotein) leads to SPG75 [114]. 

## 15. Metabolism of the Nucleotides

Purines metabolising genes lead to pure and complex HSP as neuroprotective helps in brain development. Any alterations in SPG63/*AMPD2*, SPG65/*NT5C2*, and SPG64/*ENTPD1* can sensitise neurons to various stressors [51].

## 16. Diagnosis and Its Discrepancy in HSPs

**Figure 3:**
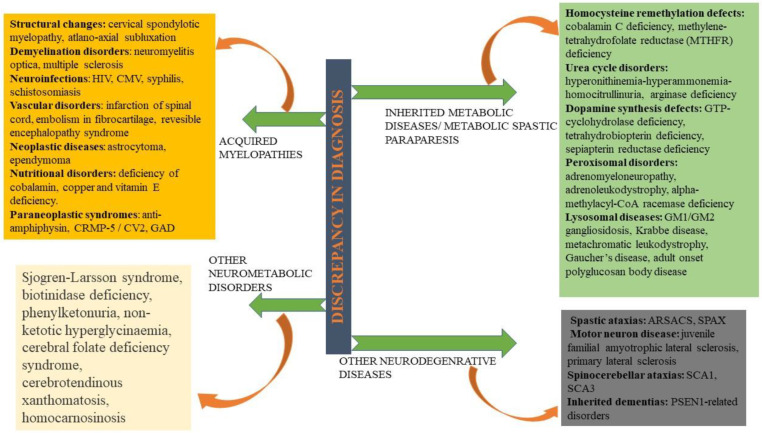
Differential diagnosis of HSP—Being an extremely heterogeneous disorder in clinical and genetic aspects, HSP is often misinterpreted with acquired myelopathies, neurometabolic disorders, inherited metabolic paraparesis, and some neurodegenerative disorders. ARSACS: Autosomal recessive spastic ataxia of Charlevoix–Saguenay; SCAs: Spinocerebellar ataxias; CMV: Cytomegalovirus; CRMP-5: Collapsin response mediator protein-5; GAD: Glutamate decarboxylase; HTLV: Human T lymphotropic virus; HIV: Human immunodeficiency virus; PSEN: Presenilin; SPAX: Spastic ataxia.

HSP is confused with several disorders, predominantly with leukodystrophies [112], primary lateral sclerosis, X-linked adrenomyeloneuropathy [128], and rarely with peripheral neuropathy, Parkinson’s, hereditary ataxia, or amyotrophic lateral sclerosis. Out of 81 HSP forms, 28 are assigned for the alternative phenotype in OMIM to complicate diagnosis (Figure 3). Specific disorder’s gene panels are used to provide an accurate clinic classification to improve diagnostic yield. 

There are different approaches available to test HSP genetically, and the most cost-effective and widely available one is the next generation sequencing (NGS), as it comprises of screening the whole exons to find the number of genes linked to HSP phenotypes but still possess limitations for differentiating the variants of large deletions, duplications, alterations in the promotor or intronic regions, and cases of triplet repeat disorders [129]. To obtain normal results, the multiplex ligation-dependent probe amplification is utilized for genes such as *SPAST* with exon deletions [130]. In a few places, first-generation sequencing is initially carried out with a set of targeted genes, and NGS is considered later [1]. The algorithm varies from clinician to clinician; some centres prefer NGS panel sequencing as a first-line investigatory protocol, even in the absence of finding a pathogenic variant. The clinician must conclude to find the gene for multiple ligation-dependent probe amplification or relate the uncertain variant with the phenotype. In addition, clinicians should have knowledge on the other monogenic diseases with the same phenotype of slowly progressing spasticity on lower limbs with no spinal cord imaging abnormalities not to be categorized in SPG. The NGS panel does not comprehensively cover HSP alone, and they include the panels for ataxias (Spinocerebellar, AR, spastic), myelination defects, and other neurometabolic disorders [1]. 

## 17. Therapeutic for HSP

Currently, no specific HSP modifying therapy is available; generally, multidisciplinary commencement is required to address symptoms such as cramps, spasms, stiffness, and deformities. To improve mobility, ankle–foot orthoses and heel raise orthotics must be acquired [1]. According to HSP *in vitro* cell line studies carried by Orlacchio et al. [131], nocodazole is seen to depolymerise the microtubules. However, more studies are required to obtain conclusive results. Few promising molecules such as tubulin-binding molecules—vinblastine, noscapine, Taxol, mitochondrial complex I inhibitors (mdvi-1), an agonist for liver X receptor (GW3965), and Glycogen synthase kinase 3 inhibitor—are shown to provide promising leads in HSP therapy by increasing the stability of microtubules and restoring the count of stable microtubules; some reverse the peroxisome transport impairment, and few alter the oxidative stress in patient-derived nerve cells and olfactory stem cells [132].

## 18. Present-Day Treatment Mode

**Figure 4:**
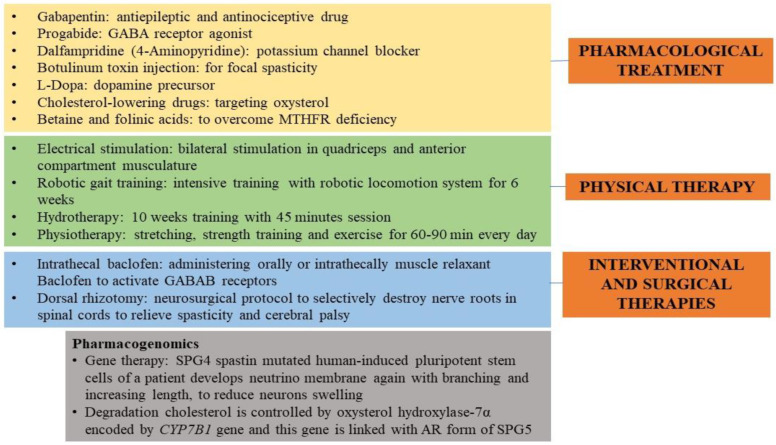
Therapies and treatment are administered to HSP individuals. The picture illustrates the pharmacological, physical, interventional, and surgical treatments given to HSP people to soothe the spasticity and improve their gait. A pharmacogenomics approach is also reported.

Capsules comprising tizanidine and baclofen are given orally as antispasmodics in HSP conditions, and oxybutynin is given to control urine urgency and infection [1]. Early intrathecal baclofen improves gait in severe spastic patients wheelchair-ridden by declining pain, disability, and muscle tone [133]. A positive response is observed in a few to 4-aminpyridine (Dalfampridine), which is awaiting conclusive results from larger group studies [134]. Botox efficacy, especially onabotulinum toxin A or botulinum toxin type-A (botulinum toxin), with stretching exercises of ankle, hips, and knees, increases gait velocity by reducing calf muscle tone to maintain strength and balance [135]. In addition, the Botox efficacy has not been accessed in the common nonmotor manifestations observed in HSP cases such as pain, fatigue, depression, and excessive daytime sleepiness, but studies by Servelhere et al. [136] found significant improvement for fatigue after Botox treatment in HSP patients by relieving spasticity and improving gait biomechanics to reduce fatigue. Yet studies are required to identify the analgesic response of the Botox in more HSP cases to obtain conclusive knowledge. Additionally, physiotherapy with a routine exercise program, especially foot and ankle orthotics and peroneal nerve stimulation, is commended for lower limb building and strength, reducing toe dragging, and enhancing the cardiovascular system’s functioning [37]. In addition, counselling and genetic assessment in patients and their families to understand the transmission risk of HSP in successive generations and the proband state (Figure 4) are recommended. Alerting clinicians while orienting and prognosing HSP as a phenotypic and genetic spectrum is strange, with extra vigilance being used for advising on SPG7 recessive forms, as a few cases can lead to AD forms [137].

## 19. Futuristic Approaches

Treatment based on the genotype has paved its way in neurological diseases in general for Huntington’s disease or spinal muscular atrophy. However, HSP is lagging due to its genetic multifariousness, mechanism diversity, various subtypes with rare forms, and slower disease progression [1]. Through gene therapy, microtubule loss can be corrected in SPG4 phenotypes [138]. Havlicek et al. [139] showed that spastin-mutated human-induced pluripotent stem cells of the patient develop neutrino membrane again with branching and increasing the length to reduce neuron swelling. Cholesterol breakdown is controlled by oxysterol hydroxylase-7α encoding the *CYP7B1*, and this gene is linked with AR SPG5.

Primarily lowering cholesterol levels is recommended for managing HSP phenotypes using atorvastatin. Deeper studies are required to obtain reliable results for utilising combined therapies [137]. The paraplegin gene mediates the fast opening of the transition pore, and it can be modulated using pharmacological protocols [1]. One study showed that intramuscular paraplegin administration in HSP mice model prevents pathology progression in neurons and protects mitochondrial structure in the peripheral nerves [140]. Gene therapy for the recessive HSP forms such as SPG11, SPG15, and SPG7 can allow editing genes at their target location or for replacement. Currently, the collaboration between world researchers and randomized control studies on large groups is required to obtain efficacious treatment for this neuromuscular pathology [1].

## 20. Conclusions

Being an extremely heterogenous malaise, HSP pathology and clinical manifestations slowly progress with higher challenges to diagnose early. In addition to the disease period’s difficulty, the proband’s genotype, age, and the complicating clinical manifestation aids to HSP severity. For the first time, we have associated genes with their chromosomal location in varying HSP subtypes through this review. In some instances, where the genes are not located yet, we have suggested a few genes with important neuromuscular function predictive of inducing HSP phenotype. These locations and chromosomes can act as clinical biomarkers to ascertain the HSP subtype and enlighten more on its pathogenesis. Works on *in vivo* and *in vitro* models can aid in analysing the pathophysiology and developing an efficacious treatment protocol.

## Figures and Tables

**Table 1:** The clinical manifestations of pure and complex HSP and a list of the prevalent clinical manifestations observed in HSP.

Pure HSP	Complex HSP
Progressive lower-extremity spastic weakness Difficulty walking—need for canes, walkers, or wheelchairs Mild diminution of lower-extremity vibration sensation Hypertonic urinary bladder disturbance Possible urinary urgency Lower-extremity paraesthesias Normal strength and dexterity of the upper extremities No involvement of speech, chewing, or swallowing Disabling symptoms without shortened life span	Impairments present in uncomplicated HSP plus other neurologic findings such as: Ataxia Seizures Intellectual disability Dementia Muscle atrophy Extrapyramidal disturbance Peripheral neuropathy

**Table 2:** Classification of HSP.

Criteria for Classification	Types
Symptoms and signs (Harding’s classification)	Pure HSP Complex HSP
Age and onset of spasticity (Harding’s classification)	Type I HSP (Early onset < 35 years) Type II HSP (Classical/late onset > 35 years)
Inheritance pattern	Autosomal dominant HSP Autosomal recessive HSP X-linked HSP Mitochondrial HSP *De Novo*
Intracellular involvement	Membrane/organelle trafficking Axonal transport Dysfunction of mitochondria Defective lipid metabolism Abnormalities in the myelination process

**Table 3:** Chromosomes, loci, and genes involved in HSPs and their subtypes.

Inheritance Mode	Chromosome Number	Locus	Genes	HSP Subtype
Autosomal dominance (AD)	1	1p31.1	NERG1 *	SPG29
	2	2p11.2 2p22.3 2q33.1 2q37.3	*REEP1* *SPAST* *HSPD1* *KIF1A*	SPG31 SPG4 SPG13 SPG30
	3	3q25.31	*SLC33A1*	SPG42
	4	4p16-15	JAKM1P1 *	SPG38
	8	8p21.1-q13.3 8q24.13	*FBXO16* *KIAA0196*	SPG37 SPG8
	9	9p13.3 9q	*UBAP1* -	SPG80 SPG19
	10	10q24.1 10q24.2	*ALDH18A1* *ZFYVE27*	SPG9A SPG33
	11	11p14.1-p11.2 11q12.3	BDNF * *BSCL2*	SPG41 SPG17
	12	12q13.3 12q23-24	*KIF5A* CKAP4 *	SPG10 SPG36
	14	14q22.1	*ATL1*	SPG3A
	15	15q11.2	*NIPA1*	SPG6
	16	16q24.3	*PARAPLEGIN*	SPG7
	19	19q13.32 19q13.33	*RTN2* *CPT1C*	SPG12 SPG73
Autosomal recessive (AR)	1	1p36.13 1p34.1 1p13.3 1p13.2 1q32.1 1q42.13 1q42.13	*ATP13A2* *HPDL* *AMPD2* *AP4B1* *DSTYK* *GJC2* *IBA57*	SPG78 SPG83 SPG63 SPG47 SPG21 SPG44 SPG74
	2	2p23.3 2q37.3	*SELENOI* *KIF1A*	SPG81 SPG30
	3	3q12.2 3q27-q28	*TFG* -	SPG57 SPG14
	4	4p13 4q25	*UCHL1* *CYP2U1*	SPG79 SPG56
	6	6p25.1 6q23-24.1	*FARS2* -	SPG77 SPG25
	7	7p22.1 7q22.1	*AP5Z1* *AP4M1*	SPG48 SPG50
	8	8p11.23 8p11.23 8p22 8q12.3	*ERLIN2* *DDHD2* *VPS37A* *CYP7B1*	SPG18 SPG54 SPG53 SPG5A
	9	9p13.3	*GBA2*	SPG46
	10	10q22.1-q24.1 10q24.1 10q24.1 10q24.31 10q24.31-10q24.33	- *ALDH18A1* *ENTPD1* *ERLIN1* *NT5C2*	SPG27 SPG9B SPG64 SPG62 SPG45
	11	11q13.1	*CAPN1*	SPG76
	12	12q13.3 12q24.31	*B4GALNT1* *C12ORF65*	SPG26 SPG55
	13	13q13.3 (TROYER SYNDROME) 13q14	*SPART* -	SPG20 SPG24
	14	14q22.1 14q24.1 14q32.31	*DDHD1* *ZFYVE26* *TECPR2*	SPG28 SPG15 SPG49
	15	15q21.1 15q21.2 15q22.31 (MAST SYNDROME)	*KIAA1840* *AP4E1* *ACP33*	SPG11 SPG51 SPG21
	16	16p12.3 16q23.1 16q24.3	*ARL6IP1* *FA2H* *PARAPLEGIN*	SPG61 SPG35 SPG7
	17	17q25.3	*PCYT2*	SPG 82
	19	19p13.2 19q12 19q13.12	*PNPLA6* *C19ORF12* *MAG*	SPG39 SPG43 SPG75
X-Linked Inheritance (XLR)	X	Xq11.2 Xq11.2 Xq22.2 Xq24-25 Xq28	MTMR8 * ZC4H2 * *PLP1* ***GLUD2*** *L1CAM*	SPG16 SPG16 SPG2 SPG34 SPG1
Mitochondrial/Maternal Inheritance	Mitochondria	- - - -	*MT-CO3* *MT-T1* *MT-ND4* *MT-ATP6*	- - - -

* The predicted genes that might induce HSP phenotypes are in bold letters.

**Table 4:** Cellular models are involved in the pathogenesis of HSP, including the genes involved and HSP subtypes in each category.

Cellular Models Involved in HSP Pathogenesis	Proteins	SPG Forms
**1. Organelle’s morphogenesis/membrane structure** Microtubule/membrane structure-associated ATPase ER tubules linking GTPase ER morphology organizer Modulating lipid metabolism and droplet formation Regenerating lysosome Adaptor proteins Tubule formation in endosomes Defects in organelles morphology inducing axonal pathology (lysosome and ER endosomes)	SPAST Atlastin SPAST Atlastin REEP1 Reticulon 2 ARL6IP1 RAB3GAP2 Protrudin REEP2 AIP4/Spartin SLC33A1 Spatacsin Spastizin AP-4 for trafficking precursors of amyloids Strumpellin REEP1 Atlastin Spastizin Spatacsin Strumpellin	SPG4 SPG3A SPG4 SPG3A SPG31 SPG12 SPG61 SPG69 SPG33 SPG72 SPG20 SPG43 SPG11 SPG15 SPG47 SPG50 SPG52 SPG8 SPG31 SPG3A SPG15 SPG11 SPG8
**2. Bone morphogenic proteins**	NIPA1 Atlastin-1 Spastin Acetyl-CoA transporter	SPG6 SPG42 SPG3A SPG4
**3. Motor proteins transportation**	KIF5A KIF1C KIF1A	SPG10 SPG58 SPG30
**4. Mitochondrial failure**	Paraplegin HSP60 IBA57 Spartin	SPG7 SPG13 SPG74/SPG77 SPG20
**5. Axon elongation path**	L1CAM	SPG1
**6. Myelination errors**	PLP1 GJC2 MAG	SPG2 SPG44 SPG75
**7. Nucleotide’s metabolism**	AMPD2 NT5C2 ENTPD1	SPG63 SPG65 SPG64

## Data Availability

Not applicable.
